# The Prevalence of *Schistosoma haematobium* and Its Impact on the Hematological Profile of Children Living in Northern Madagascar

**DOI:** 10.3390/pathogens14020172

**Published:** 2025-02-09

**Authors:** Wanesa Wilczyńska, Daniel Kasprowicz, Dariusz Świetlik, Krzysztof Korzeniewski

**Affiliations:** 1 Department of Epidemiology and Tropical Medicine, Military Institute of Medicine—National Research Institute, 04-141 Warsaw, Poland; wrichert@wim.mil.pl; 2 Clinique Medicale Beyzym, Manerinerina, Ambatoboeny District, Manerinerina 403, Madagascar; daniel.kasprowicz@icloud.com; 3 Department of Biostatistics and Neural Networks, Medical University of Gdańsk, 80-210 Gdańsk, Poland; dswietlik@gumed.edu.pl

**Keywords:** DUS, Madagascar, *Schistosoma haematobium*, schistosomiasis

## Abstract

Schistosomiasis is a parasitic disease that is considered a major threat to public health in Madagascar. The condition is endemic in more than 90% of the country’s districts. It is estimated that as much as 52% of the country’s general population is infected with *Schistosoma* spp. trematodes. The aim of the present study was to assess the prevalence values of *Schistosoma haematobium* infections in a population of children living in northern Madagascar and to determine the impact of trematode infections on the hematological profiles of the children included in the study. This screening study was conducted in 2024, and it involved a group of 170 children aged 0–17 years. The participants were required to provide urine samples for microscopic and molecular examination. The urine samples were preserved on Whatman 903 protein sever cards using the dried urine spot (DUS) sampling technique and then were transported from Madagascar to a molecular laboratory in Poland, where the samples were tested for the presence of *S. haematobium*. The present study found that the incidence of *S. haematobium* infections in the study group consisting of 170 children was as high as 67.6%. The authors observed a reduction in mean hemoglobin (Hb) and mean corpuscular hemoglobin concentration (MCHC) values in the infected children. In spite of continuous efforts to prevent the transmission of schistosomiasis in endemic countries (WHO-recommended mass drug administration campaigns), the incidence of *S. haematobium* infections was found to be exceptionally high among the study participants. *S. haematobium* infections present with a characteristic hematological picture showing signs of increased immune response and anemia. The DUS technique has been successfully used for the molecular diagnosis of *S. haematobium*. This method opens up possibilities for more effective and less expensive sample collection.

## 1. Introduction

Schistosomiasis (bilharziasis) is a parasitic disease that is caused by *Schistosoma* trematodes. In Madagascar, where schistosomiasis is endemic, the disease is caused by *Schistosoma haematobium* or *S. mansoni*. Intermediate hosts of *Schistosoma* spp. can be freshwater snails (*Bulinus* snails serve as an intermediate host for *S. haematobium,* while *Biomphalaria* snails are an intermediate host for *S. mansoni*). In fresh water, *Schistosoma* spp. eggs hatch and release miracidia (a larval stage of the parasite), which then actively search for a species-specific host (snail). Once miracidia invade a snail, they undergo further development and eventually transform into cercariae. Cercariae are shed by the intermediary host (the snail) into the external environment (freshwater bodies), where they can infect humans, who serve as the definitive host of *Schistosoma* spp. Snails can shed up to 600 cercariae daily. Transmission usually occurs when cercariae penetrate human skin during direct exposure to contaminated water, e.g., while walking barefoot in or near freshwater bodies [1,2].

Clinical manifestations of schistosomiasis can include fever, headache, muscle pain, and a rash at the site of penetration; the so-called swimmer’s itch (cercarial dermatitis). *S. haematobium* can also manifest with clinical signs and symptoms including hematuria or dysuria, and if it is left untreated, it can progress into a chronic infection, leading to bladder cancer or reproductive tract injures (in women) [3,4,5]. Schistosomiasis is a neglected tropical disease (NTD) that is endemic in 78 countries around the world, predominantly in Africa, but also in Asia and Central and South America.

In Madagascar, schistosomiasis is endemic in more than 80% of the country’s districts. The country has the fifth-highest rate of schistosomiasis in the world [6]. Urogenital schistosomiasis (*S. haematobium*) is prevalent in the northern and western parts of the island (where the temperatures often reach >28 °C, thus providing excellent breeding conditions for the intermediate host of *S. haematobium*—*Bullinus obstuscopura* snails). Intestinal schistosomiasis (*S. mansoni*) is found in the eastern, central, and southern parts of the country, i.e., in areas where the average temperatures and lower. Co-endemicity of both *Schistosoma* species is seen in the central and south-western regions of the country [6,7]. Schistosomiasis-related health problems often affect pre-school and school-age children [8,9]. The primary factors contributing to the high endemicity of *S. haematobium* in Madagascar are environmental conditions (numerous freshwater bodies that facilitate the development of the parasite), poor sanitation and hygiene conditions, limited access to clean drinking water, and low levels of health education. The difficulties in detecting *S. haematobium* within the Malagasy population are primarily associated with diagnostic limitations and insufficient healthcare funding [9]. The aim of the present study was to assess the incidence occurrence of *Schistosoma haematobium* infections in a population of children living in Northern Madagascar and to determine the impact of trematode invasions on the hematological profile of the children included in this study.

## 2. Materials and Methods

### 2.1. Study Group

**Figure 1:**
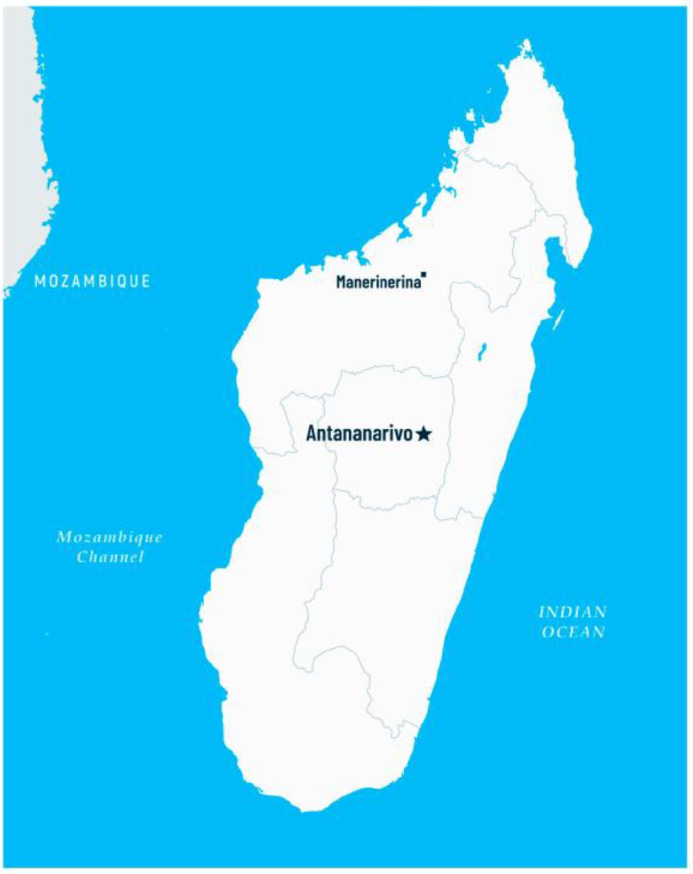
Map of Madagascar with location of Manerinerina, Ambatoboeny district (Antananarivo—the capital of the country).

The study for screening of schistosomiasis was conducted in May 2024 in the Clinique Medicale Beyzym in Manerinerina, Ambatoboeny district, Northern Madagascar (Figure 1) among 170 children (86 girls and 84 boys) aged 0–17 years (mean age 8.5 years) living in Manerinerina rural municipality. Participation in this study was voluntary and did not require the occurrence of symptoms of schistosomiasis. The only eligibility criterion was age (<18 years). The children’s parents or legal guardians were required to complete the patient form and to provide informed consent for their child’s participation in this study. In Madagascar, there exists a gender-based division of roles and tasks. Boys are predominantly involved in agricultural activities, such as assisting with land cultivation, harvesting crops, and animal husbandry. They are also more likely to engage in physical games and sports. In contrast, girls typically contribute to domestic chores (such as cooking, cleaning, and water collection) and gardening tasks (such as cultivating vegetables and gathering fruits). Girls are also more frequently engaged in craft activities (such as weaving and jewelry making) compared to boys.

### 2.2. Sample Collection

Each patient involved in this study was provided with one sterile container for a urine sample as well as instructions for sample collection. After collection, the urine samples were delivered to the medical center, where they were examined using light microscopy and then applied onto Whatman 903 protein sever cards (Whatman International, Maidstone, UK) using a sterile Pasteur pipette. Each urine sample was applied on five different discs (approximately 75–80 µL per disc). The sever cards were left in the laboratory for 24 h to dry, then they were placed into air-tight moisture absorbing bags. These prepared dried urine spots (DUS) were used for the molecular tests. After 2 weeks, the biological material was transported to Poland. Additionally, the patients included in this study underwent body temperature measurements and blood tests performed at the medical center in Manerinerina. Hematological analysis was performed on whole blood samples using the Mindray BC-3000 Plus Auto Hematology Analyzer (Mindray Bio-Medical Electronics Co. Ltd., Shenzhen, China).

#### 2.2.1. Light Microscopy

The fresh urine samples were examined for *S. haematobium* ova. The microscopic examinations were performed on centrifuged urine sediment [10] by qualified personnel from the medical center in Manerinerina.

#### 2.2.2. Real-Time PCR

Real-time PCR (qPCR) was used to identify *S. haematobium*-specific DNA sequences in the dried urine spots. For this purpose, 12 mm discs were cut out from each of the sever cards using sterile scissors. The discs were then placed in 2 mL Eppendorf tubes. Next, 1 mL of distilled water was added to each tube, and the samples were incubated at room temperature for 24 h under the same conditions as those applied by Zacharia et al. [11]. The scissors used to cut out the discs were washed with hypochlorite and distilled water after each sample in order to prevent cross-sample contamination. Nucleic acids were extracted using the Bosphore Nucleic Acid Extraction Versatile Spin Kit (Anatolia Geneworks, Istanbul, Turkey), which utilizes the silica membrane column method, in line with the manufacturer’s instructions. Real-time PCR (qPCR) assays for the detection of *S. haematobium*-specific DNA were performed using a commercial RT-PCR Kit AmpliTest *Schistosoma haematobium* (*Amplicon* sp. z o. o., Wrocław, Poland). The tests were performed using a reaction volume of 20 µL in accordance with the following thermal profile: initial denaturation at 95 °C for 5 min, 45 cycles of denaturation at 95 °C for 10 s, and annealing at 60 °C for 25 s. RT-PCR was performed using an Aria MX thermal cycler (Agilent Technologies, Santa Clara, CA, USA). A sample was considered positive when the PCR product amplification was recorded as a fluorescence increase with a Cq value ≤ 40.

### 2.3. Statistical Analysis

Statistical calculations were performed using the suite StatSoft Inc. (2014) STATISTICA version 12.0 www.statsoft.pl (accessed on 14 October 2024) (StatSoft Polska Sp. z o.o., Kraków, Poland) and an Excel sheet (Microsoft Corporation, Redmond, WA, USA). The significance of the differences between the groups of *Schistosoma*-infected vs. non-infected individuals (unpaired model) was determined with a Student’s *t*-test or Mann–Whitney U test. In all the calculations, the level of statistical significance was set at *p* = 0.05.

## 3. Results

Microscopic examinations performed on the fresh urine sediment collected from the 170 children revealed 85 cases of *S. haematobium* infections (50%). All *S. haematobium* cases were confirmed using molecular methods. RT-PCR assays for the detection of *S. haematobium*-specific DNA allowed us to identify an additional 30 infections. Thus, the overall prevalence of *S. haematobium* infections in the study group was found to be 67.7% (95%Cl: [60.67;74.73]). The infections were found to be more prevalent among the boys, and their prevalence increased with the children’s age (Table 1).

The risk for *S. haematobium* infection was found to be significantly higher in the older children (*p* = 0.0001), and the infections were mostly seen in the boys (0.0428). No statistically significant difference was found with respect to body temperature between the groups of infected vs. non-infected children (*p* = 0.3562) (Table 2).

Hematological analysis performed on whole blood samples showed lower Hb and MCHC values in the *S. haematobium*-infected individuals compared to the non-infected individuals. No statistically significant differences were found with respect to the other hematological parameters (RBC, HCT, MCV, MCH, RDW-CV) between the two groups, i.e., between *Schistosoma*-infected and non-infected children (Table 3).

## 4. Discussion

*Schistosoma* spp. infections are unevenly distributed across Madagascar. It is estimated that *S. haematobium* is endemic in the northern and western parts of the island, whereas *S. mansoni* is most prevalent in the central, eastern, and southern parts of the country [6,7]. The present research study involved children from the northern regions of Madagascar, which might explain the high incidence of *S. haematobium* infections. These findings are consistent with reports by other authors. The prevalence of schistosomiasis in Madagascar is strongly geographically correlated, and in some regions, it can reach up to 94% [12,13]. Studies by Robinson et al. [14] showed that the prevalence of schistosomiasis in Northern Madagascar was 64.5%, which is similar to the results of the present research study and comparable with the countrywide estimation of schistosomiasis prevalence in Madagascar, which stood at 52.21% [15]. Sub-Saharan Africa accounts for approximately 90% of schistosomiasis cases globally. It is estimated that in Africa alone, 112 million people become infected with bilharziasis, and an estimated 280,000 people die of *Schistosoma* spp. infection each year [16,17]. The present study showed an exceptionally high prevalence of *S. haematobium* infections in the study participants (67.6%). For comparison, the overall prevalence of schistosomiasis in children living in other African countries was found to be 15% in Nigeria [18], 6% in Tanzania [19], 45% in Gabon [20], and 29% in Ethiopia [21]. The present study also found that the prevalence of *S. haematobium* infections was higher in males compared to females; the same correlation was observed by other researchers as well [22,23]. This finding suggests that men are generally more at risk for schistosomiasis infection because of their increased exposure to contaminated fresh water, e.g., while fishing, farming, or working in irrigation systems [13,24]. Many children (particularly boys) are involved in agricultural and fishing activities from a very young age, making them equally susceptible to infection [25]. In addition, studies suggest that men are less likely to participate in MDA, which is another important factor contributing to a higher prevalence of *S. haematobium* infections in men compared to women [26,27]. A number of studies suggest that schistosomiasis can induce anemia as a result of chronic inflammation. The parasite eggs trapped inside the tissues cause irritation and bleeding from the epithelium, leading to anemia (hematuria associated with the *S. haematobium* infection may additionally aggravate anemia, as it can cause blood loss of as much as 125 mL/day); this in turn can result in iron loss of up to 37.5 mg/day [4,28]. The authors of the present study performed hematological analysis of whole blood samples collected from 170 children included in this study. The analyses showed lower Hb and MCHC values in *S. haematobium*-infected patients compared to non-infected individuals. Studies by Afrifa et al. [29] and Dejon et al. [30] showed comparable results. Low Hb concentration is an important indicator of anemia, which is a common health problem in Sub-Saharan Africa. High rates of anemia in Africa are mostly due to widespread malnutrition and high prevalence of infectious diseases that may include blood loss complications. *S. haematobium* infection is an example of such a condition, which was supported by the findings of Afrifa et al. [29] and the results of the present study. The present study found lower Hb concentrations in *S. haematobium*-infected compared to non-infected children. In addition, this study showed lower values of red blood cell parameters (HCT, MCH, MCHC), which is in line with the findings of other authors [29,31], but only the difference in MCHC values was statistically significant (*p* = 0.0015). The hematological parameters in *S. haematobium* infected patients vary depending on the intensity and duration of the infection. Schistosomiasis usually affects those living in hot, low-income countries, where the disease burden is high and where multiple infectious diseases coexist. This situation often creates diagnostic difficulties. The results of the present study provide evidence that *S. haematobium* invasions affect the hematological profile of those infected and result in a characteristic drop in RBC parameters. This finding could be helpful in the interpretation of complete blood count tests in areas where bilharziasis is endemic.

The WHO aims to eliminate schistosomiasis in all endemic countries, including in Madagascar, by 2030. Schistosomiasis control programs in Madagascar involve mass administration of praziquantel (MDA) in all school-age children [32]. The problem remains, however, that in many high-risk areas, adults are often excluded from MDA programs. There are many reasons for this situation, but the main ones include limited availability of praziquantel, a widespread belief that schistosomiasis only affects children, and a fear of possible side effects associated with praziquantel treatment, such as inability to work [33,34,35]. Consequently, adults represent a substantial reservoir of *Schistosoma* spp. and are a major source of disease transmission in local communities. A high prevalence of schistosomiasis in the adult population prevents breaking out of the vicious circle of disease transmission and successful elimination of schistosomiasis in endemic regions, and it also puts whole communities at risk of this chronic and debilitating illness [36]. Limited access to health services and a lack of quick and inexpensive diagnostic tools poses yet another difficulty in combating bilharziasis in low-resource settings [37]. Therefore, the Whatman 903 protein sever cards used as matrices for molecular diagnostics of *S. haematobium* in this study seem to be an attractive alternative to more demanding diagnostic instruments. A more comprehensive approach is necessary to achieve the WHO goal of eliminating schistosomiasis in Madagascar. This approach would primarily include educating the public about disease prevention methods (especially of the importance of personal and water hygiene) and conducting regular MDA campaigns targeting school-age children and adults alike [5].

## 5. Limitations of This Study

This study had several limitations that could have influenced the main findings. The patients were not specifically defined in terms of their health status and possible comorbidities. This study was limited by its small sample size, even though it provided preliminary data about the incidence of schistosomiasis among children living in Northern Madagascar. The available samples only allowed for the identification of *S. haematobium*, but the authors intend to continue research in this area, include more children, and expand the diagnostics to detect the occurrence of potential comorbidities, including *S. mansoni* infections.

## 6. Conclusions

The present study found that the incidence rate of *S. haematobium* infections in the group of 170 children living in Northern Madagascar was 67.6%. The analysis of the whole blood samples collected from the study participants showed lower values of red blood cell parameters (in particular, Hb and MCHC values), which was indicative of mild anemia. Molecular tests for *S. haematobium* were successfully performed on DUS that had been collected on Whatman 903 protein sever cards. Applying this method can simplify the process of sample collection and sample transportation to remote diagnostic facilities and could be a major step towards achieving the WHO’s target to eliminate schistosomiasis in African countries, including in Madagascar, by 2030. Other important interventions to eradicate the disease from Africa should also include mass drug administration to adults, implementing health awareness campaigns, and educating local people about the possible routes of schistosomiasis transmission and prevention against the disease.

## Figures and Tables

**Table 1:** Distribution of *S.ma haematobium* (light microscopy vs. real-time PCR (RT-PCR)) detected in children by sex and age (n = 170) in Northern Madagascar.

Children	No. (%) of Infections			
	Light Microscopy	95%Cl	RT-PCR	95%Cl
Total (n = 170)	85 (50.0)	[42.5;57.5]	115 (67.6)	[60.6;74.7]
Female (n = 86)	40 (46.5)	[36.0;57.1]	52 (60.5)	[50.1;70.8]
Male (n = 84)	45 (53.6)	[42.9;64.2]	63 (75.0)	[65.7;84.2]
≤3 years (n = 22)	3 (13.6)	[4.7;33.4]	9 (40.9)	[20.4;61.5]
4–6 years (n = 34)	10 (29.4)	[14.1;44.7]	18 (52.9)	[36.2;69.7]
7–10 years (n = 58)	34 (58.6)	[45.9;71.3]	43 (74.1)	[62.9;85.4]
11–13 years (n = 36)	25 (69.4)	[54.4;84.5]	28 (77.8)	[64.2;91.4]
≥14 years (n = 20)	13 (65.0)	[44.1;85.9]	17 (85.0)	[69.4;100.0]

**Table 2:** Comparative analysis of variables between infected and non-infected children (n = 170) in Northern Madagascar.

Variables	Infected Children (n = 115)	Non-Infected Children (n = 55)	Total (n = 170)	*p*-Value
Age (years)				0.0001 ^1^
Mean (SD)	9.4 (4.0)	6.8 (4.0)	8.5 (4.2)	
Range	1.0–17.0	1.0–17.0	1.0–17.0	
Median (IRQ)	10.0 (5.0)	6.0 (6.0)	9.0 (6.0)	
Sex				0.0428 ^2^
Female	52 (45.2%)	34 (61.8%)	86 (50.6%)	
Male	63 (54.8%)	21 (38.2%)	84 (49.4%)	
Body temperature (°C)				0.3562 ^1^
Mean (SD)	36.6 (0.3)	36.7 (0.5)	36.6 (0.4)	
Range	36.1–37.8	36.2–39.0	36.1–39.0	
Median (IRQ)	36.6 (0.2)	36.6 (0.3)	36.6 (0.2)	

^1^ Mann–Whitney U test; ^2^ Chi-square.

**Table 3:** Hematological parameters in infected vs. non-infected children (n = 170) in Northern Madagascar.

Variables	Infected Children (n = 115)	Non-Infected Children (n = 55)	Total (n = 170)	*p*-Value
**Hb g/dL**				0.0221 ^1^
Mean (SD)	11.1 (1.3)	11.6 (1.0)	11.3 (1.2)	
Range	7.6–15.5	9.4–14.4	7.6–15.5	
Median (IRQ)	11.2 (1.5)	11.6 (1.3)	11.4 (1.3)	
**RBC**				0.0841 ^2^
Mean (SD)	4.5 (0.5)	4.6 (0.4)	4.5 (0.5)	
Range	3.0–5.6	4.0–5.4	3.0–5.6	
Median (IRQ)	4.5 (0.7)	4.6 (0.6)	4.5 (0.6)	
**HCT (%)**				0.2441 ^2^
Mean (SD)	35.9 (3.9)	36.6 (3.0)	36.1 (3.7)	
Range	25.3–50.5	30.9–44.0	25.3–50.5	
Median (IRQ)	36.2 (4.7)	36.4 (4.2)	36.3 (4.2)	
**MCV (fL)**				0.5580 ^2^
Mean (SD)	80.0 (8.4)	79.2 (6.0)	79.8 (7.7)	
Range	51.4–105.0	64.0–93.7	51.4–105.0	
Median (IRQ)	81.2 (9.2)	78.9 (7.4)	79.6 (8.5)	
**MCH (pg)**				0.5124 ^2^
Mean (SD)	24.7 (2.8)	25.0 (2.2)	24.8 (2.7)	
Range	15.4–31.8	19.5–29.0	15.4–31.8	
Median (IRQ)	24.9 (3.4)	25.2 (3.1)	25.0 (3.3)	
**MCHC (g/dL)**				0.0015 ^1^
Mean (SD)	30.8 (1.7)	31.3 (2.1)	31.0 (1.8)	
Range	19.4–32.9	19.0–33.2	19.0–33.2	
Median (IRQ)	31.0 (1.7)	31.6 (1.5)	31.2 (1.8)	
**RDW-CV %**				0.1653 ^1^
Mean (SD)	15.5 (1.6)	15.1 (1.2)	15.4 (1.5)	
Range	12.6–21.2	13.4–19.0	12.6–21.2	
Median (IRQ)	15.3 (2.0)	15.1 (1.6)	15.2 (1.9)	

^1^ Mann–Whitney U test; ^2^ Student’s *t*-test. Hb—hemoglobin; RBC—red blood cells; HCT—hematocrit; MCV—mean corpuscular volume; MCH—mean corpuscular hemoglobin; MCHC—mean corpuscular hemoglobin concentration; RDW-CV—red blood cell distribution width.

## Data Availability

The data presented in this study are available from the corresponding author upon request.
